# RFAmyloid: A Web Server for Predicting Amyloid Proteins

**DOI:** 10.3390/ijms19072071

**Published:** 2018-07-16

**Authors:** Mengting Niu, Yanjuan Li, Chunyu Wang, Ke Han

**Affiliations:** 1 School of Information and Computer Engineering, Northeast Forestry University, Harbin 150040, China; yunzeer@gmail.com; 2 School of Computer Science and Technology, Harbin Institute of Technology, Harbin 150040, China; chunyu@hit.edu.cn; 3 School of Computer and Information Engineering, Harbin University of Commerce, Harbin 150040, China; hanke@hrbcu.edu.cn

**Keywords:** amyloid protein, random forest, RFAmy, protein classification, machine learning

## Abstract

Amyloid is an insoluble fibrous protein and its mis-aggregation can lead to some diseases, such as Alzheimer’s disease and Creutzfeldt–Jakob’s disease. Therefore, the identification of amyloid is essential for the discovery and understanding of disease. We established a novel predictor called RFAmy based on random forest to identify amyloid, and it employed SVMProt 188-D feature extraction method based on protein composition and physicochemical properties and pse-in-one feature extraction method based on amino acid composition, autocorrelation pseudo acid composition, profile-based features and predicted structures features. In the ten-fold cross-validation test, RFAmy’s overall accuracy was 89.19% and F-measure was 0.891. Results were obtained by comparison experiments with other feature, classifiers, and existing methods. This shows the effectiveness of RFAmy in predicting amyloid protein. The RFAmy proposed in this paper can be accessed through the URL http://server.malab.cn/RFAmyloid/.

## 1. Introduction

The name of the amyloid protein comes from the technique of the early immature iodine staining [1]. In many neurological diseases such as Alzheimer disease and Parkinson’s disease, large amounts of amyloid accumulation in the nervous system can be observed [2]. Many scholars believe that it may lead to degeneration or dysfunction of the brain or other organs [3,4]. At the time, the scientific community debated whether it was a matter of fat deposition or carbohydrate precipitation until finally it was discovered that it was a protein substance [5]. The exact mechanism of amyloid formation is not fully understood, but the precondition for the deposition of amyloid fibrils is the excessive production of its precursor protein [6]. The prevention of this disease should be based on active treatment of the primary disease that can induce the disease [7]. Researchers have demonstrated that the immune system has similar efficacy in humans. Therefore, to understand amyloid proteins and related diseases deeply, the most research on amyloid proteins focuses on amyloidosis [8,9,10], amyloid region [11,12], aggregation [3,13,14], and antibody amyloid [15].

Many calculation methods on the problem of amyloid accumulation have been developed, such as AmylPred [16], Pafig [17], FoldAmyloid [12], and Waltz [18]. AmylPred method mainly uses five different and independently published methods to form a consensus prediction of amyloidogenic region. Pafig employs the support vector machine to predict the amyloid protein region through the recognition of the hexapeptides associated with the aggregation of amyloid protein. FoldAmyloid realizes the prediction of the amyloid region by combining the method of predicting hydrogen bond formation with the expected bulk density of the residues. Waltz [19] employs the position-specific scoring matrix to predict the amyloid region. As reviewed [20], there are currently two major methods to study the aggregation of amyloid proteins and to identify the amyloidogenic regions that are most likely to form fibrils: (1) using a phenomenological model based on the physicochemical properties of amino acids to identify the amyloidogenic regions; and (2) modeling the microcrystalline structure of the peptides by simulating the short fibers of the amyloid fragment [21,22].

For the amyloid protein region, many studies and prediction methods exist. Garbuzynskiy et al. proposed and developed an online web server named FoldAmyloid [12]. It mainly uses the statistical data features of the amyloid protein and introduces two features, namely expected probability of hydrogen bonds formation and expected packing density of residues, to predicted amyloidgenic regions [12]. Wieczorek et al. proposed amyloid protein region prediction based on fuzzy grammar [23]. In their paper, the amyloid sequence is described by fuzzy context-free grammar and the amyloidogenic region is identified by fuzzy grammar. To accurately predict the amyloidogenic region, Emily et al. combined weighted merging of existing popular methods to create a meta-predictor called MetAmyloid [24]. There are also many methods for successfully predicting the amyloid region in amino acid sequences with computational techniques, such as biological mutagenesis and quantitative calculations. For the formation of antibody amyloid, Otoo et al. proposed the automatic and cross-species prediction method AbAmyloid [25], which employs random forest algorithm. The prediction has been tested on 12 datasets, and outperformed other methods. David et al. combined Naive Bayes and decision trees to predict amyloidogenesis in antibodies [15]. Although the mis-aggregation of amyloid may lead to some clinical studies, many studies have recently shown that amyloid still has positive significance in some aspects, for example bacterial and antimicrobial activity [4,26], fungal biofilm formation [21,27,28,29], storage of peptide hormones [3,30], the formation of zona pellucida to protect mammalian and fish oocytes [31], etc. These studies show the importance of increasing the awareness of amyloid.

Although there is a lot of research on amyloid protein, they ignored the first step of identifying amyloid protein. In this paper, we present RFAmy to identify amyloid with random forest (RF), which it based on composition and physicochemical features from protein primary sequences.

As we conclude above, machine learning frame has been employed to identify special proteins, including cytokines [32], DNA-binding proteins [33,34], RNA-binding proteins [35], lncRNA-interacting proteins [36], drug targets [37], etc. Different sequence features have been proposed to describe proteins, including Chou’s Pse features, SVMProt [38], secondary structure features [39], evolutionary features [40], etc. Different machine learning classifiers have been employed, including support vector machine [41,42], random forests [43], ANN [44], etc. There are also some special classifiers for different conditions, such as ensemble classifier [42,45,46,47,48,49,50,51,52,53,54], multi-label classifier [55,56,57,58], imbalance classifier [59,60], hierarchical classifier [61,62,63], etc. All these previous works guide us to build a frame for amyloid protein identification.

## 2. Results and Discussion

### 2.1. Measurement

To evaluate the prediction effect of the prediction algorithm used, this article selects four commonly used indicators: sensitivity (SE) (Equation (1)), specificity (SP) (Equation (2)), accuracy (ACC) (Equation (3)) and Matthew coefficient (MCC) (Equation (4)).

(1) $$\;\mathrm{SE}=\frac{TP}{TP+FN}\;$$

(2) $$\;\mathrm{SP}=\frac{TN}{TN+FP}\;$$

(3) $$\;\mathrm{ACC}=\frac{TN+TP}{TN+FP+TP+FN}\;$$

(4) $$\;\mathrm{MCC}=\frac{(TP\times TN)-\left(FP\times FN\right)}{\sqrt{\left(TP+FP)\times (TP+FN)\times (TN+FP)\times (TN+FN\right)}}\;$$

where TP indicates the number of amyloid proteins predicted in the sequence of positive cases, FP indicates the number of non-amyloid proteins predicted in the counterexample sequence, TN indicates the number of non-amyloid proteins predicted in the sequence of positive cases, and FN indicates the number of non-amyloid proteins predicted in the counterexample sequence. SE denotes the ratio of being positive in the sequence and predicting positive. SP indicates the correct rate of prediction of counterexamples.

ACC denotes the proportion of correct predictions in all the positive and negative examples, and the reliability of the MCC represents the results of the algorithm. When the difference between the positive and negative examples is large, the prediction ability can be more equitably reflected.

In this paper, the positive and negative dataset are unbalanced, so we have additionally adopted a criterion F-measure (Equation (7)) which is calculated with precision (Equation (5)) and recall (Equation (6)).

(5) $$\;\mathrm{Precision}=\frac{TP}{TP+FP}\;$$

(6) $$\;\mathrm{Recall}=\frac{TP}{TP+FN}\;$$

(7) $$\;F-\mathrm{measure}=\frac{2\times Precision\times Recall}{Precision+Recall}\;$$

### 2.2. Performance of Different Features on Cross-Validation

This section presents the selection of *n*-gram features, adaptive skip-gram features (400-D), pse-in-one features and 188-D feature to verify the validity of the 188-D and pse-in-one combined feature representation method used in this paper. The results are shown in Table 1. Here, the features representation methods that we used to compare with our feature representation method are briefly listed.

The *n*-gram features are common in natural language processing and we employed this feature in protein prediction problems [64]. This is a method of checking “*n*” consecutive words or sounds from a given text or speech sequence. The *n*-gram needs to link n words together as a feature. The *n*-gram assumes that the *n*th word is only affected by the first *n* − 1 words. The probability of the entire sentence is the product of the probability of occurrence of each word. This model helps to predict the next item in the sequence.

The Adaptive Skip-Gram Features model (400-D) is a variant of the *n*-gram model. The corpora counted by jumping a certain number or position of words were used to obtain n-gram information, Adaptive Skip-Gram features more content than n-gram. The correlation between distance and sequence amino acids to a certain extent solves the problem of feature space sparsity caused by the traditional n-gram method.

The results are shown in Table 1. In Table 1, we can see that the feature representation method used in this paper performs well on all indicators compared to other methods. The accuracy, MCC, SE, SP and F-measure all reached maximum: 89.19%, 0.739, 0.781, 0.927 and 0.891, respectively. In short, the random forest based RFAmy predictor feature extraction algorithm outperforms the others. Therefore, the feature extraction method used in this paper is feasible and effective.

### 2.3. Performance of Different Features on External Validation

To test the robustness of the proposed method, external validation is required to evaluate the developed predictive model. Therefore, we evaluated RFAmyloid on an independent dataset and again compared its performance to the performance of different feature representation methods. We only used 80% of the data to develop the predictive model, and the remaining 20% was used for external or independent verification. The independent test results are shown in Table 2. In Table 2, we can see that the feature representation method used in this paper performs well on all indicators compared to other methods. The accuracy, MCC, SE, SP and F-measure all reached maximum: 89.71%, 0.757, 0.818, 0.932 and 0.897, respectively. Independent testing confirmed the previous test results and confirmed that our proposed predictor effectively recognizes amyloid. Since the proposed method is robust in independent testing, it should be effective in predicting new amyloids.

### 2.4. Comparison with Other Classifiers

**Figure 1:**
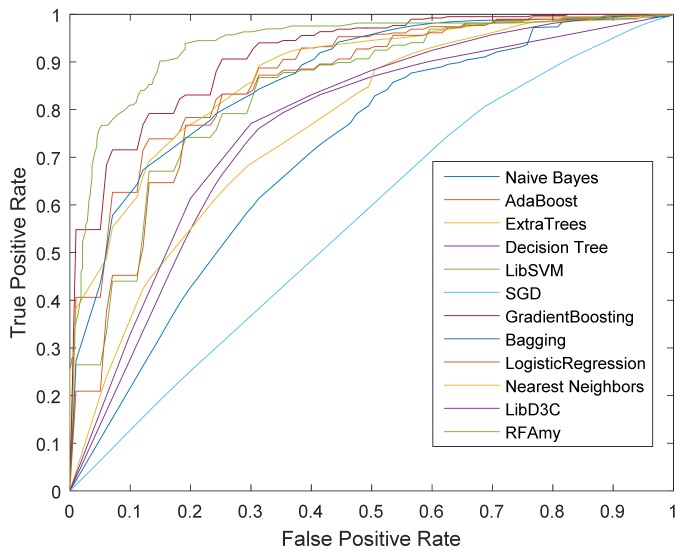
Receiver Operating Characteristic (ROC) curve for RFAmy and other methods.

In this subsection, the performance of RFAmy is compared with the performances of other classifiers, namely Naive Bayes, SGD, Nearest Neighbors, Decision Tree, LinearSVC, Logistic Regression, LibSVM, ExtraTrees, Bagging, AdaBoost, GradientBoosting, and LibD3C [65]. The experimental results are shown in Table 3. In Table 3, although the RFAmy method presented in this paper is lower than Nearest Neighbors in SP index, RFAmy is obviously superior to the four other indices. The RFAmyloid has the highest accuracy and F-measure: 89.19% and 0.891, respectively. Figure 1 shows the ROC curve (the further the curve is projected to the left, the better the effect is) of RFAmy and the comparison classifiers’ experimental results. It is well verified that the random forest classifier outperforms other classifiers in predicting the accuracy of amyloid, demonstrating the validity of the proposed method.

### 2.5. Comparison with Other Predictors

**Figure 2:**
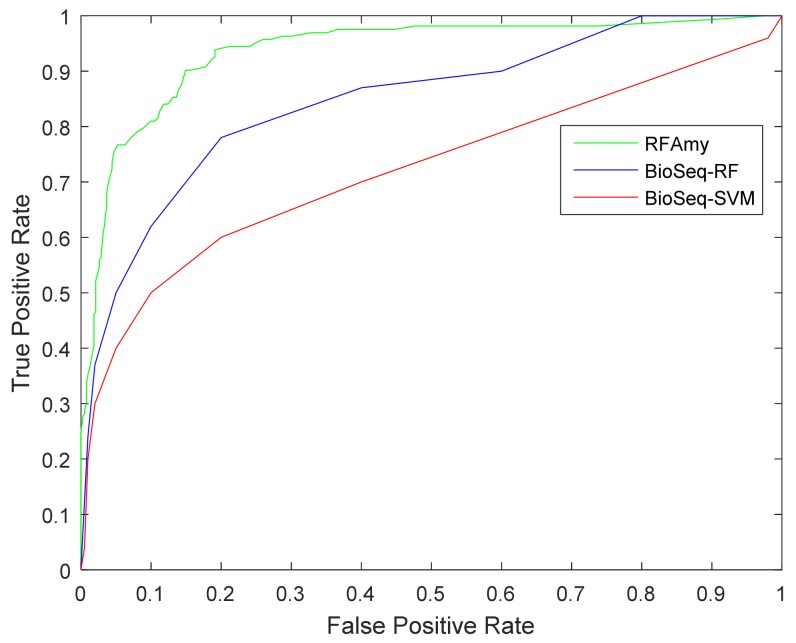
Receiver Operating Characteristic (ROC) curve for RFAmy and two other methods.

In this section, the proposed prediction method is compared with the existing prediction method BioSeq-Analysis. The online address for this method is http://bioinformatics.hitsz.edu.cn/BioSeq-Analysis/PROTEIN/Kmer/ [66]. The SVM and random forest algorithm are used in The BioSeq-Analysis prediction method. This section compars them separately. The prediction results are shown in Table 4. Figure 2 shows the roc curve of RFAmy and comparison algorithm method experimental results. Table 4 shows that the RFAmy method proposed in this paper achieved the best results on the all evaluation indicators. In addition, the ROC curve diagram (the further the curve is projected to the left, the better the effect is) shows that the RFAmy method in this paper is obviously better than the other two methods.

### 2.6. Comparison with Balanced Dataset

The results in Table 1, Table 2, Table 3 and Table 4 show that the specificity was much higher than the sensitivity, which is the effect of the unbalanced dataset in the development of predictive models. Therefore, we used a balanced dataset to develop a predictive model and compared its performance to the performance based on an unbalanced dataset. The results are shown in Table 5. From the comparison results in Table 5, we can see that, although the accuracy under the balanced dataset and the F-measure index are slightly lower than the unbalanced dataset, the sensitivity under the balanced dataset is much higher than the specificity. This also proves the importance of the selection of datasets for model prediction.

## 3. Methods

**Figure 3:**
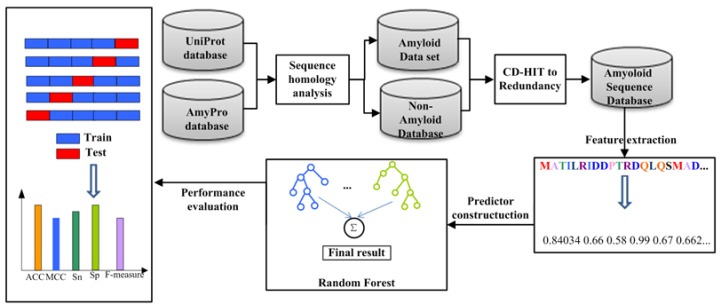
Overview of the paper framework for an Amyloid classifier. First, the original protein sequence was generated from the Uniprot and AmyPro datasets and then subjected to a de-redundant operation to generate the final protein sequence data called Amy. The second step is feature extraction of protein sequences. The third step is to use RF to classify protein sequences.

Figure 3 shows the paper framework for an Amyloid classifier. We introduce the datasets, features and classifiers in detail in this section.

### 3.1. Dataset

This study used a self-built dataset named Amy. The dataset constructin followed the common steps of protein prediction.

The first step was to search for proteins. The source databases are the Universal Protein (UniProt, http://www.uniprot.org/) and Amyloid (AmyPro, http://www.amypro.net/) [67] database. The second was to remove the sequences which are less than 50 amino acids. In the third step, protein sequences eliminated redundancy. We used the program CD-HIT to cluster proteins that meet a similarity threshold [68] and eliminate redundancy and homology biases that could lead to overestimation of performance. In this study, through these three steps, a set of amyloid data, the Amy dataset, was formed which consists of 165 amyloid proteins and 382 non-amyloid. The Amy dataset can be downloaded from the server.

### 3.2. Feature Extraction 

Feature extraction is the first and most important component in predictors [69]. We employed a multi-feature representation method that includes two feature representation methods, namely, 188-D feature extraction method based on protein composition and physicochemical properties and pse-in-one feature extraction method based on amino acid composition, autocorrelation pseudo acid composition, profile-based features and predicted structures features.

Different kinds of amino acids have their own special physicochemical properties, which can predict the type of protein as a feature of the amino acid sequence. In addition, the 20 compositional features of amino acids can describe the characteristics of the protein. Both methods achieved good predictive results. Dubchak first attempted to fuse the two features together and achieved better results in predicting protein folding patterns [9]. Afterwards, many scholars proposed a variety of feature fusion methods [70]. The 188-D combined feature extraction method extracts eight physical and chemical characteristics, the frequency of occurrence of 20 amino acids in the protein sequence, the frequency of bipartite subsequences, and the distribution of amino acids with different physical properties in the sequence. The 188-D feature is mainly obtained by the following four steps.

The first step is to extract the proportional characteristics of the amino acid components in the sequence, a total of 20 dimensions. In the second step, using hydrophilicity and hydrophobicity as an example, the compositional content of amino acids with hydrophilicity, hydrophobicity, and neutrality can be calculated to extract 3D features. In the third step, if there is a total of n hydrophilic, hydrophobic, and neutral amino acids in the sequence, calculate the proportions of the first, 25% * *n*, 50% * *n*, 75% * *n*, and the last such amino acids in the sequence of the protein in which they are located. Each category has five dimensions, so there are 15 dimensional features in all three categories. Finally, in accordance with the “hydrophilic”, “hydrophobic” and “neutral” properties, two or two combinations are constructed. The 3-D characteristics of “hydrophilic, hydrophobic,” “hydrophilic, neutral,” and “hydrophobic, neutral” are calculated, and the ratio is calculated in the sequence of the bisimplex, which is also 3D. Table 6 describes the 188-D function.

Pse-in-one has five groups of 22 features extraction methods [71]. The first group uses kmers, distance-based residue (DR), and distance pair to indicate the composition of amino acids. The second group uses auto covariance (AC), cross covariance (CC), auto-cross covariance (ACC), and physicochemical distance transformation (PDT) to represent autocorrelation features. The third group uses four indicators such as PC-PseAAC to indicate the characteristics of false amino acids; the indicators can be found in the literature [72]. The fourth group uses Top-*n*-gram, distance-based auto covariance, profile-based Auto-cross covariance, sequence conservation score, and other three indictors to represent Profile-based features. The fifth group uses secondary structure and solvent accessible surface area to represent predicted structure features. Table 7 shows the 22 feature extraction methods.

### 3.3. Classifier

For the identification of amyloid protein, random forest was selected as the classification algorithm in this study. It is popular and has been successfully used in biometrics many times [55,72,73,74,75,76]. Random forests are a combination of tree predictors. Algorithms are implemented by building multiple decision trees and using voting mechanisms to improve decision trees. Random forests are generated in the following four steps.

The first step is to generate *n* samples from the sample set by resampling. The second step is to assume that the number of sample features is *q*, and select *k* features from *q* for *n* samples, and then obtain the best segmentation point by building a decision tree. The third step is to repeat *m* times, and then generate *m* decision trees. The fourth step is to predict by a majority voting mechanism. It should be noted that, where *m* represents the number of cycles, and *n* represents the number of samples, then *n* samples constitute the sample set for training, and *m* such samples are generated in *m* cycles. 

**Figure 4:**
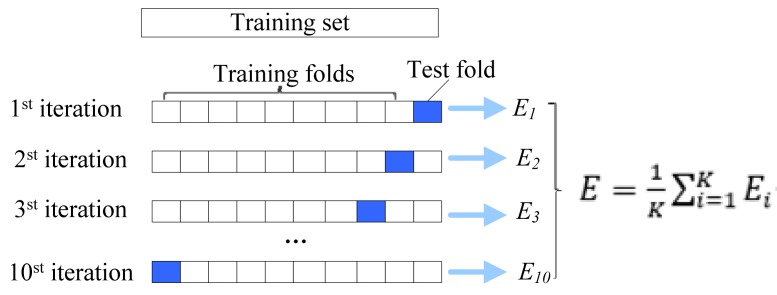
Ten-fold cross validation diagram. The dataset was divided into ten parts, and nine of them were taken as training data in turn, and one was used as test data for testing. The average value *E* of the ten-groups test results is calculated as an estimate of the model accuracy and is used as a performance indicator for the current K-fold cross-validation model. Where *E_i_* represents the cross-validation error of the *i*th group.3.4. The RFAmyloid Online Prediction Server.

In machine learning, the algorithm model needs to be trained to update each parameter in the model. Therefore, it is necessary to provide the training set as a training sample. At the same time, to describe the generalization ability of the model, a test set is needed to test and obtain the generalization error. In practical applications, cross-checking is often used as a test method because of the limited number of datasets. There are three types of cross-validation: n-fold cross-validation, folding cross-validation and independent data testing [77,78,79,80]. In three tests, the folding knife test has been widely used in bioinformatics because it produces unique results [81,82,83,84,85]. However, it takes time and resources. Therefore, in this paper, we use *K*-fold cross-validation to examine the proposed model, where *K* = 10 is the most common. In detail, the training set is divided into *K* parts, and then the *i*th is taken as the test set, the other *K* − 1 is trained as the training set. The operation diagram of the ten-fold cross-validation is shown in Figure 4. 

With the development of bioinformatics, it is important to make better use of machine learning methods to solve related biological information. As mentioned in a series of documents, the development of predictive methods and related servers is very practical and urgently needed for researchers. Therefore, based on the prediction method of the paper and the data used, we also carried out server development. The URL is: http://server.malab.cn/RFAmyloid/.

On the server, the user can paste the protein sequence or upload the file in fasta format. After submitting the protein sequence, the page will give the probability information of whether it is Amyloid protein, and query the prediction result. The dataset used in this paper can be downloaded from the web.

## 4. Conclusions

In this paper, we propose a new learning algorithm RFAmy for amyloid prediction. We used SVMProt 188-D feature representation, pse-in-one feature representation and random forest classifier. To verify the effect of the proposed predictor, we compared its performance with 10 other cross-validation and independent test sets with other feature representations. In the 10-fold cross-validation, we obtained ACC 89.19% and F-measure 0.891. In the independent test set, we obtained ACC 89.19% and F-measure 0.891. In addition, our models have better predictive effects than other feature extraction algorithms, classifiers and existing methods. The RFAmy proposed in this paper can be accessed through the URL http://server.malab.cn/RFAmyloid/. In future work, we will optimize RFAmy’s prediction performance through the improvement of feature extraction algorithms and classification algorithms. For the improvement of the classifier, the use of an integrated classifier will be considered, combining multiple classifiers to complete the classification task and improve the classification accuracy.

## Figures and Tables

**Table 1:** The result of using different feature representation methods on cross-validation.

Method	ACC (%)	MCC	SE	SP	F-Measure
188-D+Pse-in-One	89.1941	0.739	0.781	0.927	0.891
188-D	84.8482	0.626	0.655	0.932	0.626
Pse-in-one	81.31	0.5626	0.6374	0.8989	0.792
400-D	84.1105	0.634	0.691	0.917	0.838
*n*-gram (*n* = 1)	81.3187	0.522	0.534	0.930	0.802

**Table 2:** The result of using different feature representation methods on external validation.

Method	ACC (%)	MCC	SE	SP	F-Measure
188-D+Pse-in-One	89.7196	0.757	0.818	0.932	0.897
188-D	73.1841	0.524	0.512	0.960	0.678
Pse-in-one	78.7037	0.679	0.676	0.880	0.782
400-D	71.2963	0.543	0.503	0.893	0.684
*n*-gram (*n* = 1)	69.4444	0.522	0.534	0.893	0.657

**Table 3:** The result of using different classifiers based on 188-D feature.

Classifier	ACC (%)	MCC	SE	SP	F-Measure
Random Forest	89.19	0.739	0.781	0.927	0.891
Naive Bayes	75.50	0.3791	0.4606	0.8822	0.8721
SGD	77.51	0.4451	0.5515	0.8717	0.6533
Nearest Neighbors	77.70	0.4293	0.2970	0.9843	0.8818
Decision Tree	67.28	0.2567	0.5333	0.7330	0.7461
LinearSVC	77.51	0.4654	0.6242	0.8403	0.8658
Logistic Regression	79.52	0.5123	0.6545	0.8560	0.8694
LibSVM	70.02	0.0651	0.0061	1.0000	0.8239
ExtraTrees	74.95	0.4128	0.6061	0.8115	0.8087
Bagging	74.95	0.4128	0.6061	0.8115	0.7727
AdaBoost	76.78	0.4700	0.6788	0.8063	0.8763
GradientBoosting	80.26	0.5298	0.6667	0.8613	0.8668
LibD3C	86.99	0.683	0.732	0.929	0.868

**Table 4:** The result of using different methods.

Method	ACC (%)	MCC	SE	SP
RFAmy	89.1941	0.739	0.781	0.927
BioSeq-SVM	76.86	0.4419	0.4953	0.9006
BioSeq-RF	81.31	0.5626	0.6374	0.8989

**Table 5:** The result of using different feature representation methods.

Method	ACC (%)	MCC	SE	SP	F-Measure
unbalanced	89.1941	0.739	0.781	0.927	0.891
balanced	83.4962	0.757	0.847	0.823	0.865

**Table 6:** Structure of 188-D Feature.

Physical-Chemical Property	Dimensions
Amino acid composition	20
Hydrophobicity	21
Normalized van der Waals volume	21
Polarity	21
Polarizability	21
Charge	21
Surface tension	21
Secondary structure	21
Solvent accessibility	21

**Table 7:** Pse-in-one feature extraction method of protein sequences.

Category	Method
Amino acid composition	K-mer
	DR
	Distance Pair
Autocorrelation	AC
	CC
	ACC
	PDT
Pseudo amino acid composotion	PC-PseAAC
	SC-PseAAC
	PC-PseAAC-General
	SC-PseAAC-General
Profile-based features	Top-*n*-gram
	PDT-Profile
	DT
	AC-PSSM
	CC-PSSM
	ACC-PSSM
	PSSM-DT
	PSSM-RT
	CS
Predicted structure features	SS
	SASA
